# Supplementary material for: STFANet: A spatial and temporal feature aggregation network for fake face detection in videos
Source: PLoS One. 2025 Dec 10;20(12):e0337340. doi: 10.1371/journal.pone.0337340 (PMC12694830; doi:10.1371/journal.pone.0337340)
Supplement: Attachment — Submitted filename: Recommendation.pdf [file pone.0337340.s002.pdf]

Recommendation:

Accept with minor revisions (language polishing)

The manuscript is technically sound, well-structured, and presents a meaningful contribution to the field of fake video detection. The authors have adequately addressed the previously provided reviewer comments, and the experimental results are comprehensive and convincing. I recommend acceptance of the manuscript pending minor revisions, specifically related to language polishing to enhance clarity and readability.
