# Supplementary material for: STFANet: A spatial and temporal feature aggregation network for fake face detection in videos
Source: PLoS One. 2025 Dec 10;20(12):e0337340. doi: 10.1371/journal.pone.0337340 (PMC12694830; doi:10.1371/journal.pone.0337340)
Supplement: Attachment — Submitted filename: Response to Reviewers.pdf [file pone.0337340.s003.pdf]

Original Manuscript ID: PONE-D-25-07298R1

Original Article Title: "STFANet: A spatial and temporal feature aggregation network for fake face detection in videos"

To: PLOS ONE Editor

Re: Response to reviewers

Dear Editor,

Thank you for allowing a resubmission of our manuscript, with an opportunity to address the reviewers' comments. We carefully revised the language of the manuscript based on the reviewers' comments and added relevant work from the latest year for comparison.

We are uploading (A) A rebuttal letter that responds to each point raised by the academic editor and reviewer(s) ("Response to Reviewers"), (B) A marked-up copy of our manuscript that highlights changes made to the original version (as "Revised Manuscript with Track Changes"), and (C) An unmarked version of our revised paper without tracked changes ("Manuscript").

We would like to express our great appreciation to you and the reviewers for comments on our paper. If you have any questions about our response, please contact us without hesitation, and we will respond as soon as possible.

Best regards,

Guoren Yao, Gaoming Yang, Xintian Liu, Lei Chen

Reviewer #2: Recommendation:

Accept with minor revisions (language polishing)

The manuscript is technically sound, well-structured, and presents a meaningful contribution to the field of fake video detection. The authors have adequately addressed the previously provided reviewer comments, and the experimental results are comprehensive and convincing. I recommend acceptance of the manuscript pending minor revisions, specifically related to language polishing to enhance clarity and readability.

Author response: We sincerely appreciate the reviewer's positive and encouraging comments. We are grateful for your recognition of our work's technical soundness, structure, and contribution to the field of fake video detection. In accordance with your valuable suggestion, we have carefully polished the entire manuscript to improve the clarity, readability, and overall language quality. Specifically, we have refined sentence structures, corrected grammatical issues, and enhanced the flow of technical descriptions to ensure the paper reads more smoothly and professionally.

Reviewer #3: I have reviewed the revised submission thoroughly and am satisfied with the amendments made. The authors have addressed the points raised in the initial review comprehensively.

Author response: We sincerely thank the reviewer for the careful re-evaluation of our revised manuscript and for the positive feedback. We are very pleased to know that the amendments have met your expectations and that the concerns raised in the initial review have been satisfactorily addressed. We truly appreciate your recognition of our efforts and your valuable time spent reviewing our work. Your insightful comments have greatly helped us improve the quality, clarity, and overall presentation of the paper.

Thank you once again for your encouraging remarks and recommendation toward acceptance.

Reviewer #4: The authors have adequately addressed the reviewers' comments from the first round, and their responses are well-articulated and satisfactory. Besides that, I have the following comment:

The comparison with state-of-the-art methods in Table 4 lacks recent works from 2024–2025. The same applies to Table 7.

Author response: We sincerely thank the reviewer for the positive and encouraging feedback, and for carefully pointing out the need to include more recent works in Tables 4 and 7.

We fully agree with your suggestion and have revised the manuscript accordingly. Specifically, we have added a newly published 2024 work for comparison in both Table 4 and Table 7 to make the experimental evaluation more up to date and comprehensive.

As shown in references [23] and [24] below, we have introduced the most recent years' work for comparison. The results of the comparative analysis can be found in Tables 4 and 7.

[23] Xia R, Zhou D, Liu D, Li J, Yuan L, Wang N, et al. Inspector for face forgery detection: Defending against adversarial attacks from coarse to fine. IEEE Transactions on Image Processing. 2024. 33:4432-4443.

[24] Bai N, Wang X, Han R, Hou J, Wang Q, Pang S. Towards generalizable face forgery detection via mitigating spurious correlation. Neural Networks. 2025.182:106909.

We applied the code shared on github in this work to our experimental environment. The experimental environment used in this paper is as follows: The hardware platforms used in our experiments include a CPU: Intel Xeon Platinum 8255C, memory: 64G; GPU: RTX 3090, memory: 24G. The software platform consists of PyTorch 1.7, CUDA 11.0, cuDNN 8.0.5, and a Conda environment. The dataset and parameter selection are consistent with other comparison methods, and the comparison results are shown in Tables 4 and 7.

Table 4. AUC score(%) comparisons with ~~10~~12 state-of-the-art approaches on FaceForensics++ and Celeb-DF datasets.

Method	Year	Level	FF++	Celeb-DF
MesoNet [38]	2018	Video	75.30	54.80
Multi-task [43]	2019	Video	80.10	90.50
Face+Context [44]	2021	Frame	75.00	66.00
LipForensics [39]	2021	Video	97.10	82.40
Multi-attentional [41]	2021	Frame	97.60	-
FTCN [29]	2021	Video	**99.70**	86.90
Adversarial Game [42]	2022	Frame	98.41	-
M2TR [40]	2022	Video	99.51	92.93
Dual-level [32]	2023	Video	99.20	95.40
3D Residual-in-Dense [31]	2023	Video	-	92.93
Inspector [23]	2024	Frame	96.53	98.12
FIC [24]	2025	Frame	97.14	97.62
Our method	-	Video	99.33	**98.29**

Table 7. Comparison of AUC scores (%) between video-based and image-based detection models on inter-dataset experiments

Method	Year	Level	FF++(Train dataset)		Celeb-DF(Train dataset)	
			Celeb-DF	DFDC	FF++	DFDC
MesoNet [38]	2018	Video	54.80	-	-	75.30
DSP-FWA [46]	2018	Frame	64.60	-	-	72.70
Multi-task [43]	2019	Video	54.30	-	-	53.30
Capsule [47]	2019	Video	57.50	-	-	53.60
Two Branch [48]	2020	Video	73.41	-	-	-
Xception [36]	2020	Frame	65.30	-	-	-
Multi-attention [41]	2021	Frame	67.44	-	-	-
M2TR [40]	2022	Video	68.20	-	-	-
CFFs [49]	2022	Frame	74.20	72.09	-	-
Inspector [23]	2024	Frame	66.20	70.5	-	68.67
FIC [24]	2025	Frame	67.20	69.86	-	67.8
Our Method	-	Video	68.42	73.14*	66.06	70.24*

Note: The reference numbers above are consistent with those cited in the manuscript.
