# Supplementary material for: STFANet: A spatial and temporal feature aggregation network for fake face detection in videos
Source: PLoS One. 2025 Dec 10;20(12):e0337340. doi: 10.1371/journal.pone.0337340 (PMC12694830; doi:10.1371/journal.pone.0337340)
Supplement: Attachment — Submitted filename: Response to Reviewers.docx [file pone.0337340.s001.docx]

**Original Manuscript ID:** PONE-D-25-07298

**Original Article Title: “STFANet: A Spatial and Temporal Feature Aggregation Network for Fake Face Detection in Videos**”

**To:** PLOS ONE Editor

**Re:** Response to reviewers

Dear Editor,

Thank you for allowing a resubmission of our manuscript, with an opportunity to address the reviewers’ comments.

We are uploading (A) A rebuttal letter that responds to each point raised by the academic editor and reviewer(s) (“Response to Reviewers”), (B) A marked-up copy of your manuscript that highlights changes made to the original version (as “Revised Manuscript with Track Changes”), and (C) An unmarked version of your revised paper without tracked changes (“Manuscript”)*.*

We would like to express our great appreciation to you and the reviewers for comments on our paper. If you have any questions about our response, please contact us without hesitation, and we will respond as soon as possible.

Best regards,

Guoren Yao, Gaoming Yang, Xintian Liu, Lei Chen

**Journal requirements:**

**1. Please ensure that your manuscript meets PLOS ONE's style requirements, including those for file naming. The PLOS ONE style templates can be found at**

**https://journals.plos.org/plosone/s/file?id=wjVg/PLOSOne_formatting_sample_main_body.pdf and**

**https://journals.plos.org/plosone/s/file?id=ba62/PLOSOne_formatting_sample_title_authors_affiliations.pdf**

**Author response:** Dear Editor, thank you very much for your journal style requirements. First, we used the latex template provided by the official website of the PLOS ONE journal. Based on this basic template, we carefully read the template requirements you provided in

<https://journals.plos.org/plosone/s/file?id=wjVg/PLOSOne_formatting_sample_main_body.pdf> and <https://journals.plos.org/plosone/s/file?id=ba62/PLOSOne_formatting_sample_title_authors_affiliations.pdf>, and strictly followed the journal's formatting requirements.

For example, we reset the manuscript line spacing to double spacing, capitalized the first letter of the subtitle, and set the font of the Section, etc.

**2. Please note that PLOS ONE has specific guidelines on code sharing for submissions in which author-generated code underpins the findings in the manuscript. In these cases, we expect all author-generated code to be made available without restrictions upon publication of the work. Please review our guidelines at https://journals.plos.org/plosone/s/materials-and-software-sharing#loc-sharing-code and ensure that your code is shared in a way that follows best practice and facilitates reproducibility and reuse.**

**Author response:** Thank you for bringing this to my attention. We will review the PLOS ONE guidelines on code sharing and ensure that all author-generated code that underpins the findings in our manuscript is made available without restrictions upon publication. We understand the importance of transparency and reproducibility in scientific research, and we are committed to following best practices for sharing our code to facilitate reuse and further research.

**3.** **We note that your Data Availability Statement is currently as follows: [All relevant data are within the manuscript and its Supporting Information files.]**

**Please confirm at this time whether or not your submission contains all raw data required to replicate the results of your study. Authors must share the “minimal data set” for their submission. PLOS defines the minimal data set to consist of the data required to replicate all study findings reported in the article, as well as related metadata and methods (https://journals.plos.org/plosone/s/data-availability#loc-minimal-data-set-definition).**

**For example, authors should submit the following data:**

**- The values behind the means, standard deviations and other measures reported;**

**- The values used to build graphs;**

**- The points extracted from images for analysis.**

**Authors do not need to submit their entire data set if only a portion of the data was used in the reported study.**

**If your submission does not contain these data, please either upload them as Supporting Information files or deposit them to a stable, public repository and provide us with the relevant URLs, DOIs, or accession numbers. For a list of recommended repositories, please see https://journals.plos.org/plosone/s/recommended-repositories.**

**If there are ethical or legal restrictions on sharing a de-identified data set, please explain them in detail (e.g., data contain potentially sensitive information, data are owned by a third-party organization, etc.) and who has imposed them (e.g., an ethics committee). Please also provide contact information for a data access committee, ethics committee, or other institutional body to which data requests may be sent. If data are owned by a third party, please indicate how others may request data access.**

**Author response:** Thank you for your inquiry. We confirm that our submission contains all the raw data required to replicate the results of our study. We will provide the minimal data set, including the values behind the means, standard deviations, and other measures reported, as well as the values used to build graphs and the points extracted from images for analysis.

We have uploaded our code to the stable public repository of Github and provide you with the address you can access: https://github.com/yaogr56/STFANet/tree/master. We have carefully reviewed the PLOS ONE guidelines on data sharing and are committed to following these best practices to ensure the reproducibility and transparency of our research.

**4. PLOS requires an ORCID iD for the corresponding author in Editorial Manager on papers submitted after December 6th, 2016. Please ensure that you have an ORCID iD and that it is validated in Editorial Manager. To do this, go to ‘Update my Information’ (in the upper left-hand corner of the main menu), and click on the Fetch/Validate link next to the ORCID field. This will take you to the ORCID site and allow you to create a new iD or authenticate a pre-existing iD in Editorial Manager.**

**Author response:** Thank you for pointing out this issue. As required by your journal, we have registered and verified the ORCID ID of our corresponding author. In addition, we have associated our ORCID with your journal, please check it. If you have any questions, please contact us immediately.

**5. We note that Figures 1 and 2 in your submission contain copyrighted images. All PLOS content is published under the Creative Commons Attribution License (CC BY 4.0), which means that the manuscript, images, and Supporting Information files will be freely available online, and any third party is permitted to access, download, copy, distribute, and use these materials in any way, even commercially, with proper attribution. For more information, see our copyright guidelines: http://journals.plos.org/plosone/s/licenses-and-copyright.**

**We require you to either (1) present written permission from the copyright holder to publish these figures specifically under the CC BY 4.0 license, or (2) remove the figures from your submission:**

**1) You may seek permission from the original copyright holder of Figures 1 and 2 to publish the content specifically under the CC BY 4.0 license.**

**We recommend that you contact the original copyright holder with the Content Permission Form (http://journals.plos.org/plosone/s/file?id=7c09/content-permission-form.pdf) and the following text:**

**“I request permission for the open-access journal PLOS ONE to publish XXX under the Creative Commons Attribution License (CCAL) CC BY 4.0 (http://creativecommons.org/licenses/by/4.0/). Please be aware that this license allows unrestricted use and distribution, even commercially, by third parties. Please reply and provide explicit written permission to publish XXX under a CC BY license and complete the attached form.”**

**Please upload the completed Content Permission Form or other proof of granted permissions as an "Other" file with your submission.**

**In the figure caption of the copyrighted figure, please include the following text: “Reprinted from [ref] under a CC BY license, with permission from [name of publisher], original copyright [original copyright year].”**

**2) If you are unable to obtain permission from the original copyright holder to publish these figures under the CC BY 4.0 license or if the copyright holder’s requirements are incompatible with the CC BY 4.0 license, please either i) remove the figure or ii) supply a replacement figure that complies with the CC BY 4.0 license. Please check copyright information on all replacement figures and update the figure caption with source information. If applicable, please specify in the figure caption text when a figure is similar but not identical to the original image and is therefore for illustrative purposes only.**

**Author response:** Thank you very much for your attention to the copyright issues of the pictures and facial images in our manuscript. We have fully considered this issue when writing the manuscript. We assure you that there will be no infringement issues when using these facial images directly in our manuscript. The specific reasons are as follows:

(1) Legitimacy of data source: The real and fake face images we used are all from the public FaceForensics++ dataset. This dataset is an open source dataset specially built for deep fake detection research. Any researcher can obtain it for free and use it for scientific research by simply filling out the Google form provided.

(2) Permissive license: The FaceForensics++ dataset follows the MIT license, which is a more permissive open source license than CC BY 4.0. The MIT license has almost no restrictions on users, and only requires that the data source be indicated and relevant papers be cited when using the data. We have clearly marked the data source in our paper and cited relevant literature from FaceForensics++, which fully complies with its licensing requirements.

(3) Common practice in the field: The FaceForensics++ dataset is widely used as a benchmark dataset in the field of deep fake detection, and a large number of researchers directly use face images from the dataset in the images of their papers. This practice has become a common phenomenon and has not caused any infringement disputes.

If you want to learn more about the FaceForensics++ dataset, you can visit its official GitHub repository: <https://github.com/ondyari/FaceForensics>

In addition, to avoid more misunderstandings, we added explanatory statements after the titles of Pictures 1 and 2, such as: “*All involved images are sourced from the open-source FaceForensics++ dataset in the field of deepfake detection.*” and “*The example video frames are sourced from the open-access FaceForensics++ dataset.*”, which can be seen in the revised version of the paper.

**Reviewer #1: This study contributes to the field by introducing a novel approach for detecting fake faces in videos, differing from existing methods by utilizing the STFANet framework. Unlike conventional techniques, STFANet leverages a dual-path structure, which enhances the extraction and integration of spatial and temporal features. However, several areas require improvement to strengthen the manuscript.**

**1. The dataset usage is described, but the division into training, validation, and test sets needs more clarity. The authors should specify the exact number of videos used for each set to ensure reproducibility. Additionally, the study does not sufficiently address how the model performs against different types of manipulated videos, such as face-swapping, lip synchronization inconsistencies, or other deepfake techniques. A more detailed evaluation in this regard would enhance the comprehensiveness of the study.**

**Author response:** Dear reviewer, thank you for your valuable suggestions on our manuscript.

(1) First, we have made the division of training, validation, and test sets more clearly marked in the manuscript. For each dataset used for the experiment, the training, validation, and test sets are divided in a ratio of 6:2:2.

(2) The video datasets we used in our experiments are all from three public datasets: FF++, Celeb-DF, and DFDC.The following are the details of the dataset:

1) FaceForensics++(FF++): contains 5,000 videos, including 1,000 original videos and 4,000 forged videos.

2) Celeb-DF: contains 6,229 videos, including 590 original videos and 5,369 forged videos

3) DFDC* (derived from DFDC): This paper uses 5,000 videos, including 834 real videos and 4,166 forged videos.

(3) For the different types of manipulated videos you mentioned, we have verified them based on the sub-datasets of the FF++ dataset in our experiments. FF++ contains four sub-datasets, which are fake video sets generated by four algorithms: DeepFake, Face2Face, FaceSwap, and NeruralTextures.

1) DeepFake(DF): Focuses on face replacement and generates highly realistic fake videos.

2) Face2Face(F2F): Real-time synchronous replacement of facial expressions, based on 3D facial modeling and image fusion.

3) FaceSwap(FS): Generates spoof videos through face swapping, with strong controllability.

4) NeuralTextures(NT): Focuses on the texture details of characters and improves the visual quality of fake videos.

In order to ensure the details of the experiments in the paper, we have supplemented the details of the experimental settings in the experimental section so that readers can have a clearer understanding of our model and verify the effectiveness and reliability of our model.

Regarding the inconsistent lip synchronization of faces in the video that you mentioned, it is a multimodal information fusion technology, which we will consider in future research.

**2. While the model’s performance is compared with other state-of-the-art methods, statistical significance tests are missing. Conducting tests such as t-tests, ANOVA, or Wilcoxon signed-rank tests would help determine whether the observed performance improvements are statistically meaningful. Furthermore, the manuscript contains minor typographical errors (e.g., “experimens” should be corrected to “experiments”), and the overall readability would benefit from proofreading.**

**Author response:** Thank you very much for your excellent advice. It is very important to use statistical significance tests to compare the performance of different models. In order to increase the reliability of the experimental part, we perform a *Wilcoxon signed rank* test on the experimental results of the AUC scores in Table 4 to verify the effectiveness of our model. It is worth emphasizing that in this study, our model mainly verifies the detection ability of our solution through experiments, which is more practical. On the other hand, we are very sure that the use of statistical significance tests will bring more theoretical verification to our work, which will greatly promote our future work.

The modifications are as follows:

1) We added a statistical significance test: "To verify whether the observed performance differences are statistically significant, we performed a Wilcoxon signed-rank test on the AUC scores, which showed a p-value less than 0.05, indicating that STFANet significantly outperforms the other methods".

2) We changed "experimens" to "experiments".

**3. The conclusion section should more explicitly highlight the key contributions of STFANet. The advantages over other approaches should be clearly articulated, and key numerical findings (e.g., AUC scores) should be included to reinforce the model’s effectiveness. Additionally, future research directions should be elaborated, particularly regarding areas for potential improvement and further testing.**

**Author response:** Thank you very much for your constructive suggestions. According to your request, we updated the manuscript by rewriting our conclusion section to make the key contributions of STFANet more clear. In addition, we added prospects for future research work.

The modified content is as follows:

"In this paper, we proposed a face forgery detection method based on spatiotemporal inconsistent features, STFANet, which uses sampling and feature aggregation at different time steps to obtain high-quality spatiotemporal features. Additionally, the STFANet model enhanced the global dependency of feature sequences by introducing the ViT module. The experiment demonstrated the excellent detection ability of STFANet on multiple datasets. It achieved AUC scores of 99.3% and 98.29% on the FF++ and Celeb-DF datasets, respectively, which are better than the state-of-the-art methods. In the cross-dataset evaluation, STFANet showed good generalization ability. When testing the Celeb-DF dataset on the FF++ training set, the AUC score reached 68.42%."

"STFANet mainly uses the spatiotemporal features of video frames to detect face forgery, but face forgery may also involve information from other modalities, such as audio, text, etc."

In future research, we can explore multimodal information fusion techniques, such as multi-task learning, attention mechanism, etc., to integrate information from different modalities to improve the model's ability to detect face forgery."

You may also refer to our revised version to review our revisions.

**4. The introduction effectively establishes the significance of deepfake detection, but the originality of the proposed method needs to be emphasized more clearly. A direct statement on how STFANet specifically addresses gaps in existing research (e.g., "Our method resolves X and Y issues by...") would enhance the clarity of the contribution.**

**Author response:** Thank you very much for your valuable suggestions. We enhance the originality and contribution of our model STFANet by modifying the Introduction section, adding some limitations and unresolved issues of existing work, and reiterating the advantages of our model.

We updated the manuscript by adding the following:

"Although existing research has made significant progress, there are still problems such as focusing only on the spatial features of a single frame or the temporal consistency between video frames and lacking comprehensive utilization of spatiotemporal features. There are even some methods that rely on specific types of forgery techniques and have limited generalization capabilities. "

"The STFANet model can effectively integrate spatiotemporal features, and achieve robust detection of different types of deep fake videos through global feature modeling and generalization capability improvement."

**5. Figure descriptions are sometimes insufficient, particularly in distinguishing between Figure 5 and Figure 6. The differences between these figures should be explicitly explained so that readers can better understand the model’s components and processing flow. Additionally, Table 7 compares both video-based and image-based detection models, yet this distinction is not made clear in the table title or caption. Indicating whether each approach is image-based or video-based would improve the clarity of the comparison.**

**Overall, the study presents a promising approach, but these revisions are necessary to enhance its clarity, scientific rigor, and reproducibility.**

**Author response:** Thank you for your valuable comments. We are very sorry that the description of the chart was wrong due to careless typesetting. We have made modifications to the descriptions of Figure 5, Figure 6, and Table 7 based on the issues you pointed out. We have removed the inappropriate descriptions one by one and provided new descriptions so that readers can understand the comparative differences of our experimental figures more clearly.

The specific changes are as follows:

1) Change the description of Figure 5 from "The pipeline of feature aggregation" to "The structure of the Transformer Encoder". We also added graphic descriptions to Figure 5, such as "The ViT module contains multi-head self-attention (MSA) and multi-layer perceptron (MLP) modules to capture global dependencies in feature sequences".

2) Change the description of Figure 6 from "The pipeline of feature aggregation" to "The Accuracy curve obtained by training STFANet in different sub-datasets of FF++ in intra-dataset experiments".

3) Change the description of Table 7 from "AUC score (%) Comparisons with 9 state-of-the-art approaches on inter-dataset experiments" to "Comparison of AUC scores (%) between video-based and image-based detection models".

Please refer to the revised version of the manuscript for specific modification details.

Note: The reference numbers above are consistent with those cited in the manuscript.
